# SERVICE ENVIRONMENTS AND PSYCHOLOGICAL WELL-BEING AMONG RESIDENTIAL CARE FACILITY AND NURSING HOME RESIDENTS

**DOI:** 10.1093/geroni/igz038.577

**Published:** 2019-11-08

**Authors:** Yeon Jin Choi

**Affiliations:** University of Southern California, Los Angeles, California, United States

## Abstract

One’s competence, or ability to cope, becomes lower as people age due to multiple factors, such as decreased physical and cognitive functioning, social isolation, and reduced income. Considering this change, providing appropriate services may help older adults to maintain their independence and improve their psychological well-being and quality of life. Therefore, this study aimed to investigate services that are positively associated with psychological well-being (i.e., mood, psychological health, self-efficacy) of older adults. For the analysis, observations were derived from the National Health and Aging Trends Study (NHATS), which includes a nationally representative sample of Medicare beneficiaries ages 65 and older. Service environments were assessed by (1) reflect availability and usage of 13 services (i.e., service unavailable, service available/ not being used, service available/ being used by residents) and (2) number of services available within three categories (i.e., help with ADL/IADL, transportation services, social and health-related services) Both having social events and activities available and participating in it were positively associated with older residents’ mood, while using housekeeping services and walking areas (e.g., outdoor walking path) were associated with better psychological health and self-efficacy. When models were estimated with service categories, having more social and/or health-related services available were associated with better psychological health and self-efficacy. Findings of this study suggests that social events and activities, housekeeping services, and areas to walk for pleasure or exercise would improve older residents’ psychological well-being. For Not only providing those services, residential facilities should encourage older residents to participation in or use the services.

